# Left to cope alone: how pregnant and postpartum women and their newborns manage extreme heat in coastal Kilifi, Kenya

**DOI:** 10.3389/fgwh.2026.1825348

**Published:** 2026-10-12

**Authors:** Adelaide Lusambili, Fiona Scorgie, Matthew Chersich, Britt Nakstad, Stanley Luchters, Peter Khaemba, Veronique Filippi, Jeremy Hess, Sari Kovats

**Affiliations:** 1 Institute for Human Development, Aga Khan University, Nairobi, Kenya; 2 Environmental Health and Governance Centre, African International University, Nairobi, Kenya; 3 Wits Planetary Health Research Division, Faculty of Health Sciences, University of the Witwatersrand, Johannesburg, Gauteng, South Africa; 4 Public Health & Primary Care, School of Medicine, Trinity College Dublin, Dublin 2, Ireland; 5 Department of Paediatrics and Adolescent Health, Faculty of Medicine, University of Botswana, Gaborone, Botswana; 6 Division of Paediatrics and Adolescent Medicine, Institute of Clinical Medicine, University of Oslo, Oslo, Norway; 7 International Public Health, Liverpool School of Tropical Medicine, Liverpool, United Kingdom; 8 Department of Public Health and Primary Care, Ghent University, Ghent, Belgium; 9 Centre for Sexual Health and HIV AIDS Research (CeSHHAR), Harare, Zimbabwe; 10 Department of Infectious Disease Epidemiology and International Health, London School of Hygiene and Tropical Medicine, London, United Kingdom; 11 Departments of Emergency Medicine, Environmental & Occupational Health Sciences, and Global Health, University of Washington, Seattle, WA, United States; 12 Faculty of Public Health and Policy, London School of Hygiene and Tropical Medicine, London, United Kingdom

**Keywords:** coping, heat, heat coping strategies, newborn health, postpartum, pregnant, rural kenya

## Abstract

**Background:**

In coastal Kenya, high seasonal temperatures compound structural challenges that include intermittent water access, resulting in health burdens from extreme heat. Women can experience limitations in the coping strategies available to them for protecting their health and wellbeing from high indoor and outdoor temperatures. This paper, drawing on the Climate Heat and Maternal Neonatal Health Africa (CHAMNHA) study, aims to describe how mothers and their newborns in rural Kilifi cope with extreme heat in their daily routines.

**Methods:**

This qualitative study draws on key informant interviews, in-depth interviews, and focus group discussions with pregnant and postpartum women (*n* = 22), mothers-in-law (*n* = 19), male spouses (*n* = 20), community health volunteers (*n* = 22), as well as frontline health providers, policy influencers, and government stakeholders (*n* = 16). Participants were recruited in Rabai and Kaloleni Counties in Kilifi, on Kenya's coast, during a season characterised by extreme heat and drought. Data were analysed using NVivo 12, applying a combination of inductive and deductive thematic approaches.

**Results:**

Women described five main strategies for coping with extreme heat. These included reducing or removing clothing, seeking outdoor shade and fresh air, ventilating indoor spaces, bathing and drinking cold water, and reducing or rescheduling daily activities. These strategies were also adapted for infant care. Mothers reduced infants’ clothing, moved them into shaded outdoor spaces, bathed or sponged them more frequently, and used culturally familiar remedies to manage heat-related discomfort and rashes. These practices provided immediate but partial relief. Heat-retaining and poorly ventilated housing, unreliable electricity, water scarcity and contamination, and diminishing tree cover limited women's options. Some strategies also introduced additional risks. Sleeping outdoors increased exposure to mosquitoes and personal-safety concerns, while opening windows brought dust into homes. Expectations concerning modesty, women's domestic workloads, and appropriate newborn care further constrained coping and sometimes created tension with older household members. Coping therefore involved continuous trade-offs between short-term thermal relief and social, environmental, and health risks.

**Conclusion:**

Pregnant and postpartum women in rural Kilifi manage extreme heat through individual and household practices shaped by cultural expectations and material constraints. These practices offer only temporary relief, increase caregiving demands and, in some cases, shift rather than reduce risk. Responsibility for adaptation therefore falls largely on women themselves. Protecting maternal and newborn health is likely to require heat–health guidance in routine antenatal and postnatal care, engagement of households and community actors, and investment in reliable water, cooler housing and shade.

## 1 Background

Extreme heat has intensified across many regions as the global climate continues to warm. For pregnant and postpartum women, heat exposure coincides with the physiological demands of pregnancy, childbirth and recovery, alongside the practical demands of caring for newborns and other household members (1–3). Exposure to high ambient temperatures during pregnancy has been associated with an increased risk of preterm birth, low birth weight, stillbirth and hypertensive disorders of pregnancy (4, 5). Heat also has important impacts on wellbeing, including through dehydration, fatigue, sleep disruption, anxiety and physical discomfort during pregnancy and the postpartum period (1–6). Furthermore, newborns are vulnerable to heat risks since they have limited physiological capacity to regulate body temperature (3, 6–8).

A small but growing body of research has documented strategies used by pregnant and postpartum women to manage heat. Existing evidence from West, East and Southern Africa suggests that pregnant and postpartum women and caregivers rely on a broadly similar set of immediate, low-cost practices to reduce heat exposure and discomfort (9–12). Ethnographic research in Burkina Faso, for example, found that caregiver strategies relevant to infant heat protection included placing babies in shaded areas, reducing layers of clothing and wrapping, and timing infant care activities to cooler periods of the day (9). Pregnant women in the same setting similarly adjusted daily activities to cooler hours, reduced physical exertion and described cold water as necessary for relieving thirst despite concerns that it might lead to a “big baby” (9). In The Gambia, pregnant and postpartum women engaged in agricultural work used comparable strategies, including shifting farm work to cooler times of the day, seeking shade during breaks, pacing or slowing work when possible, and increasing fluid intake when available (10). Evidence from Kenya similarly shows that pregnant women remained responsible for domestic and caregiving tasks despite high temperatures, with limited household support restricting their ability to rest or reduce heat exposure (2). More recent Photovoice research in rural Zimbabwe documented a similar repertoire, with women resting under trees, verandahs and improvised shelters, cooling themselves with wet cloths and by wetting the ground before lying on it, using clay pots and woven covers to keep drinking water cool, and laying infants undressed on mats in the shade or breastfeeding them there, while continuing domestic and agricultural work through the hottest hours (11).

Across these African settings, women's capacity to cope with extreme heat was shaped by material resources, livelihood demands, household position and cultural expectations. In Burkina Faso, coping was constrained by limited household resources, unsuitable housing and caregiving responsibilities (9). Similarly, poverty, food insecurity, gendered labour expectations and a lack of alternative livelihoods prevented women farmers in The Gambia from reducing heat exposure or resting during pregnancy (10). Heat-retaining housing, diminishing tree cover and unreliable water access constrained women's coping in Zimbabwe (11). Women's capacity to adapt was also unequally distributed. For example, in The Gambia, older or higher-status women had greater freedom to interrupt work during extreme heat, while younger and economically disadvantaged women more often continued working through the hottest hours (10). Practices such as resting, seeking shade or reducing workloads therefore reflected not individual choice alone, but women's material circumstances, their standing within households and communities, and the expectations of those around them.

A similar pattern is evident in South Asia. Evidence from Sindh, Pakistan, illustrates both the use and the limits of behavioural change to cope with heat. There, the White Ribbon Alliance documented how pregnant and postpartum women and health providers attempted to manage heat exposure through informal strategies, including increasing their fluid intake (12). A policy dialogue report from India found that pregnant women living in hot urban environments sought shade, changed the timing of activities and attempted to improve airflow in their homes (13). The evidence from Africa and South Asia suggests that heat-coping practices among pregnant and postpartum women are often similar and largely behavioural, while the capacity to use them is shaped by social, material and structural conditions. In addition, the effectiveness of these practices in both settings was constrained by factors beyond women's immediate control. These include housing and the built environment, limited cooling infrastructure, and the conditions under which women had to travel and carry out daily activities.

Broader heat-coping research among the general population reinforces this pattern. In urban households in Sekondi-Takoradi, Ghana, residents relied mainly on accessible, low-cost coping measures, including opening windows and doors for ventilation, adjusting daily routines, reducing clothing and bedding, using water for cooling, sleeping outdoors and, where affordable, using electric fans (14). These practices extended to care-giving, including the use of damp cloths to cool children. However, the use of fans was often constrained by power outages and high electricity costs (14).

Evidence among female farmworkers in Central Florida found that heat-coping strategies among women of reproductive age included drinking water or fluid-and-electrolyte-replenishing drinks such as Gatorade, wearing wide-brimmed straw hats, taking breaks when possible and seeking shaded work areas (15). However, these practices were severely constrained by workplace conditions beyond workers’ control. Production quotas linked to piece-rate wages discouraged breaks, limited toileting facilities led some women to deliberately restrict water intake despite recognising the risks, and enclosed greenhouse environments made cooling difficult (15).

Awareness of heat-related health risks among pregnant and postpartum women also remains uneven. Research suggests that many women do not recognise extreme heat as a threat to their own health or that of their infants (16), while access to heatwave warnings and weather information may be limited by gendered patterns of information access and communication (17). Thus, women's capacity to cope is shaped not only by their material circumstances and ability to act, but also by their awareness of heat-related risks and access to relevant information.

Although existing studies across a range of settings describe a set of heat-coping behaviours among pregnant and postpartum women, important gaps remain in understanding how these strategies are enacted and constrained in everyday life. In particular, little is known about how women implement heat-coping strategies for themselves across pregnancy, childbirth and the postpartum period, while simultaneously acting to protect their newborns from heat. We also lack evidence on how coping is shaped by interacting influences and constraints at individual, household, and community levels, and how these interactions play out in East Africa, especially coastal Kenya. As part of the Climate Heat Maternal and Neonatal Health Africa (CHAMNHA) consortium's wider programme of research, this paper addresses these gaps by documenting how women cope with heat during pregnancy, childbirth and the postpartum period, both for themselves and their infants. Specifically, it explores how women manage heat within the home, including adjustments to physical activities, and how infant-care practices are modified during periods of high temperature.

## 2 Methods

### 2.1 Study design, population

We employed a descriptive qualitative design incorporating in-depth interviews (IDIs), focus group discussions (FGDs), and key informant interviews (KIIs). The study population included pregnant and postpartum women, mothers-in-law, and male spouses to recently pregnant women, community health volunteers (CHVs), and key stakeholders in the community including community leaders, traditional birth attendants, religious leaders, and government officials from the ministries of health, water, agriculture, environment and local administration. The study was conducted in Rabai and Kaloleni sub-counties of Kilifi County, coastal Kenya. The sites were selected because Aga Khan University had an established programme of research in the area, residents had identified extreme heat as a concern, and the region experiences sustained high temperatures, above 23°c. A detailed description of the social context of Kilifi is provided in the CHAMNHA series of papers (17–20).

### 2.2 Sampling and recruitment

Participant recruitment was facilitated through established Aga Khan University community networks. Women were eligible for inclusion if they were at least 28 weeks pregnant or between 4 and 12 weeks postpartum (Table 1). Mothers-in-law were included if they were currently living with a pregnant or postpartum woman or had done so within the previous year. Community health volunteers (CHVs) were recruited if they had worked in this role for a minimum of two years and were actively engaged with pregnant and postpartum women in their communities. Key informants were purposively selected based on positions that provided insight into the study communities and the issues under investigation.

**Table 1:** Eligibility criteria by participant group.

Participant group	Method	Included if	Excluded if
Pregnant women	IDI	≥28 weeks’ gestation, verified by a healthcare worker; primigravid or multigravida	Under 28 weeks’ gestation, or gestation not verifiable
Postpartum women	IDI	4 to 8 weeks after childbirth; facility or home birth	Outside the 4 to 8-week window
Mothers-in-law	FGD	Shares a homestead with a woman who gave birth in the past 2 years	No such recent birth in the homestead
Male partners	FGD	Primary partner of a woman in the homestead who gave birth in the past 2 years	Not the primary partner of the woman concerned
Community health volunteers	FGD	More than 2 years in the CHV role; identified through Community Unit Heads	2 years or less in the role
Key informants	KII	TBA, Community Health Committee member, religious leader, village chief or head, or community public health officers, and leaders of various key ministries like agriculture, gender, water, reproductive rights etc	Prior IDI or FGD participant in this study; not in a listed role

Participants included pregnant and postpartum women (*n* = 22), mothers-in-law (*n* = 19), male spouses (*n* = 20), community health volunteers (*n* = 22), as well as frontline health providers, policy influencers, and government stakeholders (*n* = 16). Table 1 below summarises the inclusion and exclusion criteria.

### 2.3 Ethics clearance and consenting process

Ethical approval for this study was obtained from the Aga Khan University Ethics Committee (Ref: 2020/IERC-94 [v2]); the National Commission for Science, Technology and Innovation (Ref: BAHAMAS ABS/P/20/7568); the London School of Hygiene & Tropical Medicine Research Ethics Committee (Ref: 22685); and the Kilifi County Government (Ref: DOM/KLF/RESCH/vol.1/66).

Researchers approached consent in ways that respected variations in literacy levels and local norms of trust and communication. For example, rather than treating consent as procedural formality, researchers created space for dialogue, explaining the study in accessible language and, where necessary, in local Giriama dialect with support from Community Health Volunteers (CHVs), and allowing participants to raise questions. Literate participants reviewed the information sheets before signing once satisfied with the content. For participants unable to read, the same information was shared verbally in the local language, with a trusted witness such as a spouse or CHV present to ensure transparency and support comprehension. These participants provided a thumbprint, witnessed by a CHV to confirm that consent had been freely given. Signed consent forms were stored securely in a locked cabinet at the AKU field office data manager in Kilifi, with access restricted to authorised study staff. The forms will be retained in accordance with institutional data-retention requirements.

### 2.4 Data collection

Fieldwork was carried out by AL and PK, with some support from SC (named in Acknowledgements) who had experience working with coastal communities.

The study team translated and back-translated study interview guides from English into Swahili to ensure that questions were clear, culturally appropriate, and easy to understand. Tools were piloted before data collection, which led to changes in how heat was described in the guide; the full procedure is reported elsewhere (3). In-depth interviews and focus group discussions were held in places familiar to participants, including rooms within or near health facilities, open verandas, waiting areas, in their own homes and shaded outdoor spaces. These locations were chosen to reflect where people normally talk, wait, and rest, helping conversations feel natural rather than formal. Key informant interviews were conducted in offices or work settings that were convenient for participants.

Nine focus group discussions were held, three each with mothers-in-law (*n* = 19), male spouses (*n* = 20) and CHVs (*n* = 22), most of which were held at the health facilities. In-depth and key informant interviews were conducted individually and audio recorded, with the interviewer keeping detailed field notes that were shared and discussed daily among the research team. Background information was collected from all participants. Interviews lasted for approximately one hour while group discussions ranged from one to two and a half hours. Participants were reimbursed transportation costs and refreshments were provided during group discussions as part of respectful engagement.

### 2.5 Data management, transcription and analysis

A professional transcriber and his team transcribed the interviews and focus group discussions verbatim and translated interviews conducted in Swahili into English, consulting the study researchers where clarification was required. AL and PK audited transcription quality by listening to approximately 10% of the recordings alongside the corresponding transcripts to check accuracy and consistency. Recordings, transcripts and field notes were stored on password-protected study computers.

We followed Braun and Clarke's thematic analysis framework (21). Analysis began during data collection. AL, PK and FS reviewed field notes and discussed emerging patterns, then open-coded a small sample of transcripts, combining deductive codes derived from the study objectives and interview guides with inductive codes from participants’ accounts. These codes were discussed, refined and organised into a codebook that guided coding of the remaining transcripts in NVivo 12. Coding and interpretation remained iterative. The wider CHAMNHA team met with researchers every two weeks to discuss emerging findings and compare interpretations, while AL, PK and FS grouped related codes into broader categories and themes and compared findings across IDIs, FGDs and KIIs to identify recurring patterns and differences between participant groups.

For this paper, AL undertook a focused analysis of all coded material and quotations relating to heat coping. This involved returning to the relevant transcripts and coded extracts, comparing accounts across participants and data sources, and refining the themes most relevant to how women managed heat for themselves and their infants. AL discussed these interpretations with the wider research team over several months, and the team agreed on the final themes presented in this paper.

## 3 Results

In Kilifi County, pregnant and postpartum women described the everyday labour involved in coping with rising heat. Managing their bodies in settings with high temperatures was physically demanding due to the physiological changes associated with pregnancy and childbirth, combined with the continued necessity of performing daily chores.

Decisions about where to rest, how to stay cool, and how to care for themselves and their newborns were made within a broader context of local housing design involving traditional and modern materials, material scarcity, and drought conditions. Most women relied on simple practices such as pouring water over their bodies and bathing, but water sources were often unreliable or contaminated. As the homes did not have active cooling measures such as fans, the women sought outside areas of shade as relief from the heat. Outdoor shade was increasingly scarce, however, as natural tree cover had diminished due to ongoing deforestation. These practices provided only short-term relief. Table 2 below outlines the main components of these findings, including the specific activities undertaken, their intended outcomes, and their unintended consequences.

**Table 2:** Pathway summary of heat coping strategies.

Coping strategies	Pregnant & postpartum strategies	Infant care strategies	Intended outcomes	Unintended outcomes
1. Clothing adjustment	Wearing light, loose clothing; removing undergarments; sleeping partially or fully unclothed at night	Dressing infants in light, clothing; temporary uncovering during peak heat (no full undressing)	Cooling and comfort; reduced sweating, rashes, and irritability; improved rest	Insect exposure; tension with norms of modesty (women) and with cultural limits on infant undressing
2. Seeking outdoor shade and fresh air	Sitting under trees; resting or sleeping outdoors, including at night	Taken outdoors for shade, settling, and sleep	Improved airflow; thermal relief; rest and sleep	Mosquito exposure; risk to personal safety and inability to use mosquito nets outdoors
3. Cooling indoors	Opening windows and doors; sitting near doorways; using shaded compounds	Indirect cooling through caregiver actions	Reduced indoor heat; increased airflow	Dust exposure
4. Bathing and drinking	Frequent bathing; drinking cold water; use of ice packs or cold compresses	Bathing or washing to relieve heat and rashes; sponging in early days and supplemental feeding in infants	Rapid internal and external cooling; hydration; soothing and rash relief	Use of limited household water supply
5. Reducing Intensity and adjusting the timing of daily activities	Scheduling chores and water collection to cooler hours	Reduced heat exposure; more caregiving time with infants	Lower heat strain; protection during pregnancy and postpartum recovery	Fatigue shifted to early morning or night

### 3.1 Clothing adjustment

Pregnant and postpartum women described reducing clothing as one of the most immediate and accessible ways of coping with intense heat. Women spoke of heat as a deeply embodied experience, managed through ongoing adjustments to what they wore as temperatures rose and fell throughout the day. As one pregnant woman explained, “*When the temperatures are very high, you look for a lighter cloth and wear it*” (IDI, Pregnant woman).

Pregnant women explained how heat was so overwhelming that it disrupted rest entirely, leaving night time clothing intolerable. One woman described, “*When I experience heat, I don't sleep with clothes on. I just remain this way*,” gesturing to her bare skin (IDI, Pregnant woman).

Postpartum women described similar adjustments, “*When I came from the hospital it was hot, I was sweating, my breasts were sweating. I had to remove my blouse, my bra to breastfeed my baby who was also sweating.”*

Community Health workers repeatedly observed these practices and described them as widespread rather than exceptional. A public health officer noted, “*No bra, no underpants, no petticoat… most of them stay bare, maybe only with a shawl. You will find they are not clothing the baby especially on the upper side… they will tell you, ‘The child is feeling hot.’”* (KII, Public Health Officer).

Similarly, a sexual and reproductive health officer described encountering pregnant women without underwear during clinic visits, explaining that, “*You may find the pregnant woman… when you tell her to climb on the bed, she does not have a panty. When you ask them, they say that they sweat*” (KII, SRH Officer). Healthcare workers informed researchers that clothing removal was becoming a socially normalized response to heat; it also brought consequences, including increased exposure to insects and the psychological discomfort of being partially unclothed in shared spaces. Our findings showed that women were prioritizing physical relief over modesty, even in clinical settings where bodily coverage is typically expected.

Pregnant and postpartum women described carefully modulating infant clothing throughout the day, reducing layers during peak heat and covering babies again as temperatures dropped. One woman explained, “*When heat increases… I try to reduce the clothes so that he [baby] can get some fresh air, then I cover him again*” (IDI, Pregnant woman).

Key informants confirmed that reducing infant clothing was a common, everyday response, often used to manage heat rashes. As a religious leader observed, “*You will see child pass with their chests bare… when a parent realizes the child is getting hurt by the heat, the safest way is to remove the clothes so the baby can be cool*” (KII, Religious Leader). This practice sat uneasily alongside a local expectation that young infants be kept covered for fear of witchcraft.

### 3.2 Seeking outdoor shade and fresh air

Women seek shade because indoor temperatures in their dwellings are too uncomfortable. During the day, shade from trees provided the most convenient solution. One pregnant woman noted, “*I cannot rest inside (house); I usually sit under a tree… I take my mat and rest under it (tree),…inside the house is like the body is burning*” (IDI, Pregnant woman).

Caregivers described taking babies outside when they became restless indoors, associating fresh air with comfort and relief from heat. A postpartum woman explained, “*Now you are inside the room and the baby is asleep… if you don't take him outside, he cries. My mother told me to take him (baby) out, he doesn't want to stay there (inside the house)*” (IDI, Postpartum woman).

Mothers-in-law reinforced this practice, advising that babies should be kept in shaded outdoor areas during hot weather to avoid heat-related skin problems: “*During the day when it is hot, you don*’*t expose the baby to extreme heat… You take it outside into the shade*” (FGD, Mother-in-law).

At night, however, the situation became more complex and dangerous. The high indoor temperatures did not subside after sunset, and for many women, sleeping indoors remained unbearable. This led some to continue using outdoor spaces at night, even though they recognized the risks. A pregnant woman from Rabai described the difficult choice involved: “*You have to get outside at night, and this place is scary since it is a remote area*” (IDI, Pregnant woman). Health workers confirmed that night-time outdoor sleeping had become common when indoor heat was intolerable. A religious leader noted, “*There is heat at night too… people sleep with their windows open and sometimes sleep outside*” (KII, Religious Leader), while a County Medical Officer explained, “*Sleeping outside… becomes one of the coping mechanisms (for mothers) whereby they want to avoid the heat from their houses*” (KII, Medical Officer).

Many choose to sleep outdoors despite the unsafe locations, security risks, and increased exposure to mosquitoes. The risk of malaria is much greater for infants. One mother linked her baby's illness directly to night-time exposure:

“When my baby got malaria… it was from sitting under that tree at night.” Community health volunteers described how difficult it is to ensure families use insecticide-treated bed-nets: “I tell them not to sleep outside because they can't use mosquito nets there” (FGD, Male CHV).

Mothers-in-law informally explained to the researchers that seeking outdoor shade is not a simple preference, but a response to limited resources and few alternatives. During the day, outdoor shade provides a relatively safe way for women and infants to cope with heat. At night, however, the same strategy exposes families to health and safety risks.

### 3.3 Cooling indoors

Ventilating the home emerged as one of the most common ways women attempted to cope with indoor heat. Opening windows or doors was intended to release trapped heat, improve airflow, and create a more tolerable space for resting, breastfeeding, and caring for newborns. However, women's ability to ventilate their homes was shaped by both the design and material structure of housing and cultural expectations around newborn care. Several homes lacked windows altogether, reflecting the type of traditional housing used by the community.

In some households, opening windows was discouraged due to long-standing beliefs that cold air or outdoor dust could harm a newborn. These beliefs placed women in a difficult position, where their efforts to relieve heat could be interpreted as careless or risky. One mother described this tension clearly: “*I had to open the window because the house was dusty and hot in the night and the baby was crying. When the baby developed a red skin, my mother-in-law said it was because I opened the window and that I shouldn't have opened [it].*” Decisions about ventilation are therefore not individual choices but relational ones, negotiated within family hierarchies, where women may be blamed by older women when infant health concerns arise.

Researchers observed that a small number of households benefited from better-designed homes. As one pregnant woman noted, “*In our village the houses have been built with good ventilations to let fresh air in*.” Yet such examples were rare and cooling depended largely on maximizing natural airflow or resorting to manual fanning. In this setting, electric fans were very uncommon, and even when available, electricity was unreliable.

Health workers highlighted the importance of nature-based solutions in the surrounding environment when it came to reducing indoor heat. As one health officer observed, “*Most of them in town have fans… but here, if we have more trees planted in the homestead, you will not feel more heat*” (KII, Health Officer). A community health worker explained “*mothers in those homes with at least some drought resistant trees can get shade and some relief, and if there was a way to for the elders to encourage everyone to plant trees, we can all beat heat”.* This points to the role of housing design and tree cover as culturally acceptable, low-cost forms of protection in this setting. Overall, ventilating the built environment reveals how heat coping is shaped by social norms, housing inequality, and environmental conditions, underscoring the need for structural solutions rather than reliance on individual adaptation alone. As one mother said, “*Provide electricity so that those who can afford fans or air conditioners have the choice to stay cool.”*

### 3.4 Bathing and drinking

Bathing in and drinking cold water emerged as among the most central and instinctive ways women in rural Kilifi managed heat across pregnancy and into the postpartum period. Women described drinking, bathing, and using cold water to soothe their babies as everyday practices woven into daily life, shaped by bodily experience, shared knowledge, and limited access to other forms of cooling. In the absence of electricity, fans, or reliable indoor cooling, water became one of the few available tools for managing heat.

Postpartum women described drinking cold water frequently, including during the night, as a way of cooling their bodies and relieving discomfort. One woman explained, “*I used to drink a lot of water even at night, I could wake up at night and just drink water. Personally, I take water more frequently*” (IDI, Postpartum woman). Key informants described similar patterns among pregnant women, noting how the need to consume cold water could become intense. A sexual and reproductive health officer observed, “*…drinking cold water is there even now. There is a pregnant woman where I live in Mariakani who wakes me up every morning asking for cold water, and even in the evening when it is very cold, she says she is feeling hot*” (KII, SRH).

While women experienced drinking cold water as internally cooling, elders and community leaders expressed concern about possible harm to the unborn baby. A religious leader explained, “*She could drink very cold water till my mother complained that it could affect the baby… she may think she is escaping from the extreme heat, yet she is hurting the unborn baby*” (KII, Religious Leader). These accounts show how women's attempts to manage heat were closely watched and sometimes contested by more powerful figures in the household, creating tension between the desire for bodily relief and the need to observe cultural norms at this time.

Cold water bathing was also described as a primary coping strategy, particularly among pregnant women. Bathing occurred multiple times a day and, at times, during the night as heat intensified. One pregnant woman explained, “*You will be forced to wake up and take a shower*” (IDI, Pregnant woman). Health workers confirmed how widespread this practice was, noting, “*The majority of the women… usually bathe very often*” (FGD, Female CHV). An environmental officer linked frequent bathing to the management of heat rashes, which were worsened by pregnancy-related sweating: “*After some few hours, you will find her in the bathroom… she has those heat rashes and the temperatures are too hot, so she keeps showering*” (KII, Environmental Officer). Here, bathing functioned both as a cooling method and as a response to relieve physical symptoms.

Mothers described bathing babies frequently to reduce heat, calm irritability, and manage rashes. One postpartum woman explained, “*I would wash the baby to reduce the extreme temperatures*” (IDI, Postpartum woman). Community health volunteers reinforced this observation, stating, “*Whenever given or bathed with cold water, the baby relaxes “* (FGD, Male CHV). A public health officer similarly observed, “*…the temperatures are too hot that you have to keep on showering the baby to contain the baby's temperature*” (KII, Public Health Officer). A religious leader summarized this pattern seasonally: “*During every hot season, babies will have rashes and they are washed more often… their mother washes them often to cool the extreme heat*” (KII, Religious Leader).

In some cases, caregivers also gave small amounts of water to newborns to drink, to calm crying or heat-related distress. While not universal, this practice appeared across accounts: “*…water is given to their newborn babies to soothe them and stop them from crying*.” Community health workers and elders noted that even small amounts of water seemed to relax overheated infants.

We found that not all households used water in the same way. In some households, early newborn bathing was avoided due to postpartum beliefs about vulnerability and protection. As one community health officer explained, “*They believe a baby should not take a shower for seven days… so they sponge bathe or urinate on the baby to cool the temperature*” (KII, Community Health Officer). Some caregivers described using locally available substances to cool infants when heat caused discomfort or rashes. These practices were understood as protective and culturally appropriate responses to heat. As one key informant explained, “*They wash the child with salted water… others make concoctions out of leaves and use the residue to wash the child*” (KII, Chief).

Water scarcity is a defining feature of daily life in Kilifi, and women in our study spoke at length about the challenge this posed to their efforts to cope with the heat. They noted that frequent bathing and drinking were shaped by limited and unreliable water access, with boreholes serving large populations and becoming strained during extreme heat. As one woman noted, “*a single borehole can sustain 700 people.”*

These practices highlight how decisions about using water to cope with heat are shaped by cultural understandings of newborn care, purity, risk, and resource scarcity. In other words, bathing and drinking are not simply acts of hygiene or hydration. They are socially and culturally shaped responses to extreme heat, built into caregiving roles, but buffered by cultural beliefs and material constraints. While water-based strategies offer immediate relief for women and infants, they also generate new risks, tensions, and uncertainties.

### 3.5 Reducing intensity and adjusting the timing of daily activities

To manage heat, many women described adjusting the timing and intensity of their daily activities, carefully deciding what to do, when to do it, and what to pause altogether, in order to protect both their bodies and their babies’ bodily health.

Some women shifted to lighter domestic work in the heat of the day, while others stopped working altogether. One postpartum woman explained, “*I was just [doing] light duties… so that when I deliver, I get good results*,” framing reduced labour as a deliberate form of (self-) care. Another woman, who was pregnant at the time, described a similar response, saying simply, “*When the sun gets hotter, I do not go on with work*.” These decisions show how women actively monitored heat and adjusted their activities to avoid exhaustion and overheating, even when such choices went against cultural expectations that they should continue doing household work.

Reorganising daily tasks also helped protect babies from unnecessary exposure to heat. Physically demanding activities such as fetching water, which often involve long walks in the sun, were shifted to early morning hours when temperatures were lower. Even then, women reduced the amount of work they did. One postpartum woman reflected, “*Previously I could go four trips in the morning, in the evening three trips, but I have reduced that now*.” Reducing the number of trips meant spending less time walking in the heat and conserving energy for caregiving.

However, researchers were also told informally that such adjustments were practiced by only a few women, as cultural expectations largely positioned women as responsible for completing domestic tasks regardless of heat, pregnancy, or recovery. Although practiced by a minority, this form of task-shifting functioned as a quiet but effective form of care, revealing how women are trying to navigate cultural expectations while seeking to protect their own health and that of their babies in environments with limited alternatives.

## 4 Discussion

Women’s accounts, together with insights from their support networks and key stakeholders, reveal that coping with heat in Kilifi is a deeply embodied and relational process negotiated through domestic routines, and shaped by cultural expectations and material scarcity.

### 4.1 Clothing reduction

Reducing clothing was one of the most immediate forms of heat coping, consistent with findings from The Gambia, Burkina Faso and Pakistan (2, 9–12). Our findings, however, show that this was more than a simple response to discomfort. Women progressively removed clothing as heat intensified, including overnight and during clinical encounters, while repeatedly adjusting infant layers throughout the day. This response was negotiated against norms of bodily coverage. In the Gambian study, women wore lighter clothing at home but felt unable to do so in the fields because of norms around dress and modesty (10), suggesting that norms relax in private spaces but hold in public ones. An earlier analysis of the same CHAMNHA dataset described women sweating and wishing to “strip naked but cannot”, and a community leader noted that “the baby must be totally covered” (3).

Our findings suggest that intense heat can push these boundaries further, including into clinical spaces where greater bodily coverage is normally expected. Some community members may read such undress as neglect or poor hygiene rather than as a coping strategy with consequences for how women are received in care and viewed in the community. In these settings, clothing reduction does not negate heat exposure, however, it redistributes risk. Reduced clothing increased exposure to insects and, where mosquito nets were avoided because of heat, potentially increased vulnerability to malaria. Progressive undressing may therefore signal not effective coping, but the limits of individual coping in persistently hot homes and care environments.

### 4.2 Seeking shade outdoors for rest and sleep

Women frequently moved outdoors to seek shade, airflow, and relief when indoor spaces became unbearable, particularly during the night. This strategy reveals an important contradiction whereby the same behaviour that provides thermal relief can create additional health and safety risks. Studies from Uganda and Tanzania link outdoor sleeping with increased mosquito exposure and malaria risk (22–24), while heat is a commonly reported reason for non-use of mosquito nets (3, 25). Mothers therefore managed competing risks of reducing immediate heat exposure while potentially increasing infectious-disease and night security risks for themselves and their infants. This may constitute maladaptation (26), where coping with one climate-related risk generates another. Night-time outdoor sleeping therefore highlights the limits of household coping when housing fails to provide thermally safe indoor spaces.

### 4.3 Cooling indoors

Opening windows and doors was a common response to indoor heat, yet ventilation is neither universally available nor simply an individual choice. Windowless and poorly ventilated homes, unreliable electricity and limited access to fans restricted women's ability to cool indoor spaces. Importantly, ventilation was also socially negotiated. Newborn care across sub-Saharan Africa has long emphasised protection from cold air and draughts, which are widely understood as causes of infant illness (27, 28). Women therefore navigated competing understandings of thermal safety: opening a window could relieve heat but expose them to blame from the elders if an infant subsequently became unwell. These findings show how rising temperatures disrupt established newborn thermal-care norms and necessitate new intergenerational negotiations over appropriate care. As seen by our findings, ventilation practices expose structural inequalities in heat protection, leaving women to manage indoor heat through constrained behavioural adjustment rather than effective environmental adaptation.

### 4.4 Household water strategies

Cold water, whether used for drinking, bathing or soothing infants, was central to coping with extreme heat in Kilifi, consistent with water-based strategies reported among pregnant women in The Gambia and Burkina Faso (9, 10). In high-income countries, however, such strategies are generally supported by reliable water supplies and indoor cooling systems (30, 31). In Kilifi, water was one of the few cooling options available to households without electricity or fans, yet access became less reliable during extreme heat because of drought-related shortages (32) and increased pressure on boreholes. Similar constraints have been reported in Burkina Faso and Zimbabwe (9, 11).

Water use also reflected the interaction between embodied experience and cultural meaning. Women described cold water as internally cooling and reported drinking it frequently, including at night. During pregnancy, however, this practice was monitored and sometimes challenged by older relatives and religious leaders who believed that it could harm the unborn child: “She may think she is escaping from the extreme heat, yet she is hurting the unborn baby.” Women in Burkina Faso were similarly advised to avoid cold water in late pregnancy because “it can make the child bigger” and delay labour, potentially leading to caesarean section. Mothers-in-law sometimes enforced this restriction, while some women were advised to drink hot water even during extreme heat (9). Our findings demonstrate that the feasibility and acceptability of water-based cooling depend not only on the availability of water but also on whose body is being cooled, at what stage of the life course and under whose authority. This supports anthropological argument that water's social and material dimensions are “separated only by convention” (33) and extends it to heat adaptation, where the same cup of cold water may be understood simultaneously as relief for the pregnant woman and a risk to the fetus.

Related ideas about protection shaped the use of water in newborn care. Many mothers bathed their infants frequently to reduce heat, calm irritability and relieve rashes. Some households, however, avoided bathing newborns during the first seven days because of beliefs about protection. This differs from the pattern reported across much of sub-Saharan Africa, where early bathing is common and delayed bathing is more often attributed to cold weather or health-worker advice than to culturally informed ideas of protection (34). Avoiding bathing did not necessarily mean avoiding cooling. Caregivers who withheld full baths continued to cool infants through other practices such as sponging and using urine on the baby to cool the temperature, using salted water or leaf preparations. Washing and cooling were therefore understood as related but not interchangeable forms of care. Although World Health Organisation (WHO) guidance recommends delaying a newborn's first bath for at least 24 h to prevent hypothermia (35), its application in hot environments must also account for caregivers’ concerns about heat and the alternative cooling practices they may adopt.

Some caregivers also gave newborns water to sip “to soothe them and stop them from crying.” Similar practices have been documented in West Africa and South Asia (29), and our findings extend this evidence to coastal Kenya. WHO advises that infants under six months should not be given water “even on hot days” because it can reduce breastfeeding and increase the risks of diarrhoea and malnutrition (36). These risks may be heightened in settings where water is scarce or unsafe. Heat-health guidance should therefore promote more frequent breastfeeding as the appropriate response to infant thirst and heat exposure while acknowledging the embodied observations and caregiving concerns that lead families to offer water.

Our findings position water as both a material cooling resource and a culturally meaningful medium of protection, comfort and healing. They echo anthropological accounts of water's longstanding association with healing and raise the question of “who can legitimately interpret water-related embodied experiences” (33). Water-based practices cannot therefore be understood solely as individual behavioural choices: they are shaped by water insecurity, household authority, reproductive status and culturally embedded interpretations of maternal and infant wellbeing. Interventions should engage respectfully with these understandings rather than dismissing them as misconceptions, while also addressing the structural water insecurity that limits women's capacity to protect themselves and their infants during extreme heat. Future research should examine how local understandings of water's role in healing, protection and caregiving influence the acceptability and adoption of water-based cooling guidance.

### 4.5 Adjusting the timing of daily practices

Women adjusting their work schedules, reducing tasks, shifting labour to early hours, or stopping entirely during peak heat was another coping strategy. This is consistent with findings from The Gambia, India, Pakistan, Burkina Faso and Zimbabwe showing how pregnant women are modifying agricultural and domestic workloads despite persistent social expectations to (8–12). Importantly, the constraint in our study was not lack of awareness. Women monitored heat and understood the benefits of reducing exposure, yet such adjustments remained uncommon because domestic responsibilities persisted regardless of pregnancy, postpartum recovery or temperature. The ability to cope therefore depended partly on women's control over their time and labour. The CHAMNHA community-based behaviour-change intervention project mobilised household and community support networks to assist mothers and their infants to protect themselves from extreme heat. Where adjustments were made, reducing water-collection trips lowered heat exposure while also reducing the water available to the household precisely at the time when hydration needs were greatest, a risk documented previously in this setting (37). Women were better able to protect themselves and their infants when male partners and mothers-in-law shared water collection, household work and childcare. Yet support was uneven because men undertaking domestic work could face social disapproval, while women whose partners worked away had fewer opportunities for assistance. Heat protection is therefore relational and shaped by household labour arrangements and gender norms. Interventions focused solely on changing maternal behaviour may have limited effect where the underlying distribution of domestic labour remains unchanged, and evaluations of such interventions should report who benefits from them rather than only outcomes such as average uptake.

## 5 Contributions of this study

This study extends existing research by showing that heat coping during pregnancy and the postpartum period is not simply a set of individual behavioural responses, but an embodied, relational and socially negotiated process. By examining clothing reduction, outdoor shade and sleeping, indoor ventilation, water use and adjustments to daily activities together, it shows how women continually balanced immediate thermal relief against other health and social risks. Removing clothing, sleeping outdoors, avoiding mosquito nets, opening windows, giving newborns water and reducing water-collection trips could each relieve heat, but could also increase exposure to mosquitoes and malaria, night-time insecurity, social judgement in the community and in care settings, blame from elders if an infant fell ill, the risk of infant diarrhoea, or household water shortage. Coping may therefore redistribute or generate risk rather than remove it, pointing to maladaptation at household level and to the limits of individual coping where housing, electricity and water supply fail.

The study also makes a distinctive contribution by examining maternal and infant heat protection together. Women's ability to protect themselves and their infants depended less on awareness of heat risk than on their control over time, labour and household resources, and was shaped by household authority, gendered divisions of labour, cultural understandings of maternal and newborn safety, and unequal access to water, adequate housing and mechanical cooling. The same practice could carry different meanings depending on whose body was being cooled and at what reproductive stage as cold water could represent relief for a pregnant woman, danger to the fetus, or comfort and protection for an infant. Rising heat is also unsettling newborn thermal-care norms and guidance built largely around protecting infants from cold, requiring new intergenerational negotiations over clothing, ventilation and bathing. By bringing embodied experience, cultural meaning and material constraints into the same analysis, the study provides new qualitative evidence from coastal Kenya. Effective heat adaptation must also engage household members and community norms, support a fairer distribution of domestic labour, address water insecurity and inadequate housing, and adapt newborn care guidance to hot settings.

## 6 Strengths and limitations

By combining interviews, FGDs, and key informant perspectives, we captured both personal and structural dimensions of heat exposure that are often overlooked in public health research on maternal health. We found that including multiple community actors alongside women, from mothers-in-law and male spouses to CHVs, health workers, local leaders, and health managers, strengthened credibility and revealed the intergenerational dynamics that are shaping coping practices. As one of the earliest studies linking heat to maternal and newborn experiences in this region, it brings valuable insights for informing climate-sensitive health interventions. Its rural, low-resource setting, where access to electricity, mechanical cooling and reliable water is limited, shows how adaptation unfolds when behavioural and water-based strategies are among the few options available.

The study also has limitations. Data were collected during a specific period of high temperatures and may not capture seasonal or year-to-year variation in coping. The focus on rural Kilifi means that the findings may transfer less readily to settings with different ecological or cultural contexts. Some practices were described by key informants and family members rather than by women themselves, and may reflect their interpretations of women's behaviour. Finally, we did not collect environmental or physiological measurements, so reported experiences could not be linked to measured heat exposure or health outcomes.

## 7 Recommendations

While qualitative and context-specific, our findings suggest several implications for similar low-resource, hot settings. Newborn care guidance, which has largely focused on preventing hypothermia, may benefit from complementary advice on safe infant cooling during extreme heat, developed and tested with caregivers and clinicians. Given that some caregivers gave water to young infants to relieve heat-related distress, breastfeeding counselling could encourage more frequent feeding in hot weather while engaging respectfully with the reasons caregivers offer water. Where feasible, improving ventilation and thermal comfort in maternity and postnatal facilities, and ensuring that women who reduce clothing during care because of heat are met with respectful, non-judgemental care, may support both comfort and care-seeking. Because women's coping practices may be monitored, contested or enabled by others, heat-health interventions are likely to be more effective if they engage mothers-in-law, male partners, religious leaders and community health volunteers, and support household members in sharing domestic tasks, particularly where women lack household support. At the policy level, our findings indicate that water security and thermally safe housing warrant consideration as components of maternal and newborn heat protection, and that drought and heat early-warning information could be linked to maternal and newborn health services. Future research should combine qualitative accounts with measured exposure data across seasons, examine the safety and acceptability of locally practised infant-cooling strategies, and assess not only overall intervention uptake but also who is able to access, adopt and benefit from such interventions.

## 8 Conclusions

Pregnant and postpartum women in rural Kilifi coped with extreme heat through multiple incremental adjustments: reducing clothing, seeking shade and fresh air, ventilating their homes, drinking and bathing in cold water and rescheduling daily tasks, and they adapted many of these practices to cool their infants. These practices reflect were negotiated with others and constrained by their surroundings. Elders, mothers-in-law, partners and religious leaders monitored, contested or enabled women's choices, while homes retained heat, water was scarce and sometimes contaminated, electricity was unreliable and tree cover was diminishing. Women recognised the risks of heat, but their ability to act on this knowledge depended on their control over time, labour and household resources. Several practices brought only temporary relief, added to women's caregiving demands or created new risks, from exposure to mosquitoes and household water shortages to the giving of water to young infants. Rising heat is also unsettling newborn care norms built largely around protection from cold. Protecting mothers and newborns from extreme heat therefore cannot rest on individual women alone. Our findings suggest that this requires engaging households and trusted community actors, supporting shared domestic labour, adapting newborn care guidance to hot settings and integrating heat–health advice into routine antenatal and postnatal care, and, where feasible, investing in reliable water access, thermally safe housing, accessible shade and climate-responsive health facilities. In this way, responsibility for staying safe in extreme heat can be shared by the households, communities and systems around women, rather than borne by women alone.

## Data Availability

The datasets generated or analysed during this study are not publicly available because full transcripts could enable people familiar with the study sites to identify participants. They are available from the corresponding author on reasonable request.
